# Adaptive surgical approach to giant inguinoscrotal hernia with enterocutaneous fistula in an adult paraplegic male with severe lordoscoliosis causing loss of domain: A case report and review of literature

**DOI:** 10.1016/j.ijscr.2025.111576

**Published:** 2025-06-27

**Authors:** Jack L. Knott, William J. Ennis, Pier Cristoforo Giulianotti, Chandra Hassan

**Affiliations:** a University of Illinois Chicago College of Medicine, Department of Surgery, Wound Healing & Tissue Repair, Chicago, IL, United States of America; b University of Illinois Chicago College of Medicine, Department of Surgery, General, Minimally Invasive, & Robotic Surgery, Chicago, IL, United States of America

**Keywords:** Giant inguinoscrotal hernia (GIH), Case report, Enterocutaneous fistula, Paraplegia, Lordoscoliosis, Loss of domain

## Abstract

**Introduction:**

Giant inguinoscrotal hernias (GIH) are rare and can be complicated by loss of domain (LOD), which limits standard surgical repair options due to increased intra-abdominal pressure (IAP) and the risk of post-operative respiratory compromise. No prior reports describe GIH with LOD in patients with severe spinal deformity.

**Presentation of case:**

A 34-year-old paraplegic male with severe lordoscoliosis and bilateral strangulated GIH presented with small bowel strangulation and underwent emergent bowel resection. Due to LOD and anterior peritoneal displacement from scoliosis, standard abdominal reintegration was impossible, and the hernia was not reduced. His post-operative course was complicated by a scrotal enterocutaneous fistula. Two years later, bowel evisceration from the ECF site prompted elective reconstruction. Intraoperatively, a continuous chamber between the abdomen and scrotum was identified, containing most bowel loops, including the fistulized segment. The fistulized segment was resected, and remaining loops repositioned into the scrotum, which functioned as a permanent neoperitoneal cavity. Postoperative edema resolved conservatively, and the patient resumed normal bowel movements.

**Discussion:**

This case highlights the challenges posed by severe spinal deformities in GIH management. Traditional reduction strategies to mitigate IAP following full bowel reintegration were impractical due to anatomical constraints. Instead, the preserved scrotal sac served as a functional extension of the abdominal cavity.

**Conclusion:**

This is the first reported case of bilateral GIH with LOD in a paraplegic patient with severe scoliosis. The novel approach of scrotal repositioning offers a viable alternative when standard abdominal reintegration is unfeasible.

## Highlights

• Giant inguinoscrotal hernias (GIH) are hernias that extend below the midpoint of the thigh.
• Loss of domain can occur in patients with decades-long hernia chronicity or anatomical abnormalities
• Elevated intraabdominal pressure may result following attempted hernia reduction into the abdominal cavity
• A paraplegic male with severe lordoscoliosis presented with GIH and subsequent scrotal enterocutaneous fistula formation
• Intraoperatively, the scrotum was utilized as a neo-peritoneal cavity to permanently house repaired bowel.

## 1 Introduction

Inguinal hernias range in severity from asymptomatic, reducible hernias in the groin to giant inguinoscrotal hernias (GIH), which extend beyond the midpoint of the thigh [1]. Albeit rare, GIHs are more common in rural areas and often present after decades-long neglect [2]. While most cases can be managed electively, delayed presentation increases the risk of incarceration, strangulation, and loss of domain (LOD) [[3], [4], [5]]. Without prompt intervention, these complications can lead to bowel obstruction, ischemia, and perforation, necessitating emergent intervention [[6], [7], [8]].

Surgical repair remains the definitive treatment for inguinal hernias, with a variety of approaches available based on surgeon preference and patient anatomy [9,10]. However, in patients with LOD, standard reduction and closure techniques pose risks of increased intra-abdominal pressure (IAP) and postoperative pulmonary dysfunction [11]. Proposed techniques to mitigate increased IAP include the creation of a pneumoperitoneum, debulking of abdominal contents through resection, Stoppa's technique, and the use of scrotal skin flaps, but no standardized method exists [4,[11], [12], [13]]. Additionally, no case reports document GIH in patients with LOD and severe spinal deformity.

Here, we highlight the complex management of a 34-year-old paraplegic male with severe lordoscoliosis and a strangulated, incarcerated, bilateral GIH, small bowel perforation, and subsequent enterocutaneous fistula (ECF) formation. We describe an adaptive surgical approach utilizing the scrotum as a neoperitoneal cavity for bowel repositioning following ECF takedown.

This case report has been reported in line with the SCARE checklist [14].

## 2 Case presentation

A 34-year-old male with paraplegia from T6-T7 gunshot wounds, severe lordoscoliosis, neurogenic bladder status post-cystectomy and ileal conduit, sacral decubitus ulcers, and anemia presented to our tertiary medical center's emergency room with a large, bilateral inguinoscrotal hernia, severe scrotal pain, and erythema.

**Fig. 1:**
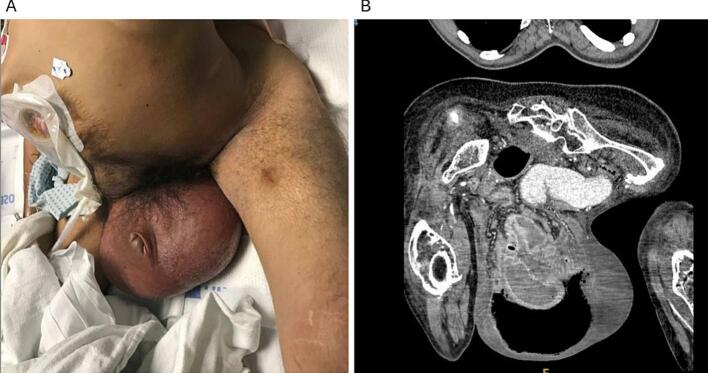
A) Giant inguinoscrotal hernia on presentation to the emergency department demonstrating erythema and buried penis. Ileostomy is present in the right upper quadrant. B) Computerized topography scan of the abdomen and pelvis remarkable for dilated bowel loops and free air in the scrotum.

Physical examination revealed an edematous, erythematous, irreducible scrotal hernia with buried penis (Fig. 1A). Computed tomography (CT) imaging demonstrated a bilateral inguinal hernia with thickened, dilated small bowel loops and free air in the scrotum, concerning for small bowel strangulation and spontaneous perforation (Fig. 1B).

The patient underwent emergent exploratory surgery via an inguinal approach, revealing a strangulated right inguinal hernia with perforated bowel. The gangrenous small bowel segment was resected and left in discontinuity for a planned second look. Two days later, scrotal cavity washout and small bowel anastomosis were performed; however, due to severe scoliosis and loss of abdominal domain, bowel reduction into the peritoneal cavity was not possible. A wound vacuum-assisted closure (VAC) device was applied over 6-in. Vicryl mesh to cover the scrotal defect.

**Fig. 2:**
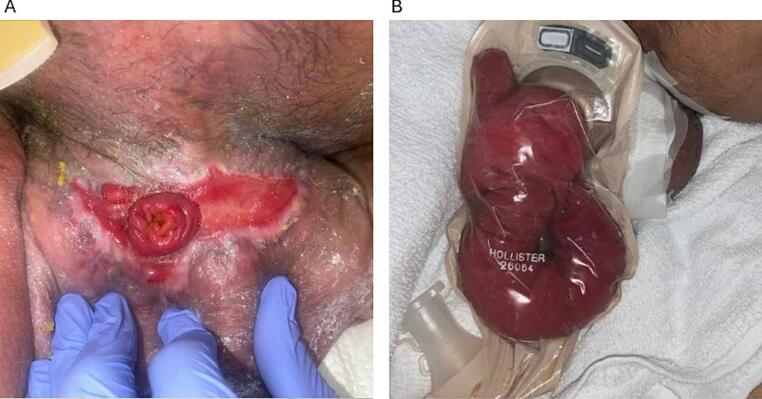
A) Scrotal enterocutaneous fistula surrounded by intact, epithelialized skin. B) Eviscerated bowel from former ECF site contained within an ostomy device.

Postoperatively, the patient developed recurrent scrotal bleeding and a high-output enterocutaneous fistula (ECF) at the anterior right scrotum. The patient was started on total parenteral nutrition (TPN) and managed with an ostomy appliance, averaging 1600 cc fistula output daily. During his two-month hospitalization, the scrotal wound re-epithelialized, leaving only the scrotal ECF exposed (Fig. 2A). Multiple surgical reevaluations under anesthesia were conducted, but given adequate wound healing and the patient's high surgical risk, the procedure was abandoned in the OR and conservative management was continued.

Two years later, the patient returned with a partial bowel evisceration from the ECF site (Fig. 2B). He expressed his inability to live in his current condition, prompting an elective surgical consultation for ECF takedown and wound closure. Considering his severe lordoscoliosis, loss of abdominal domain, large scrotal hernia with high-output scrotal ECF, prolonged TPN effects, and presence of an ileal conduit, he faced a high risk of complications, including bleeding, infection, and even death. Despite these risks, the patient was determined to proceed due to his profound impairment in quality of life.

**Fig. 3:**
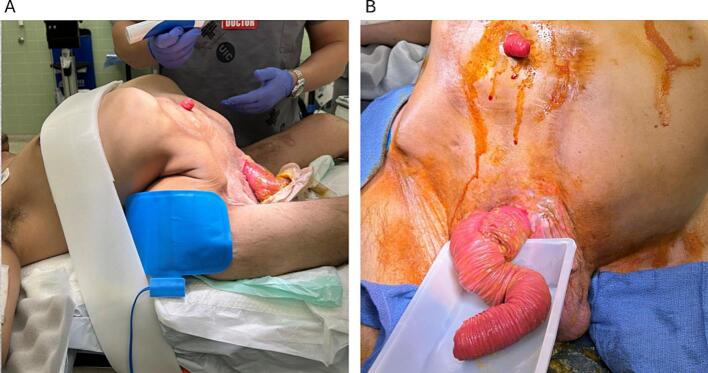
A) Patient positioned supine on the operating table, with severe lordoscoliosis and loss of domain visible. B) Eviscerated bowel segment from former ECF site.

Under general anesthesia, the patient was positioned supine with consideration for his anatomy (Fig. 3). A midline vertical incision was made from the xiphoid process, revealing a peritoneal cavity occupied by proximally displaced abdominal organs. The hernia was overriding the pubis, and the anatomy of the groins was subverted. The stomach extended to the pubic area, and the aorta and retroperitoneum had shifted anteriorly, nearly abutting the anterior abdominal wall. The hernia sac extended continuously from the peritoneal cavity into the scrotum, with no identifiable inguinal canal between the abdomen and scrotum. Most bowel loops, including the fistulized segment, were contained within the scrotum. Minimal adhesions were identified.

**Fig. 4:**
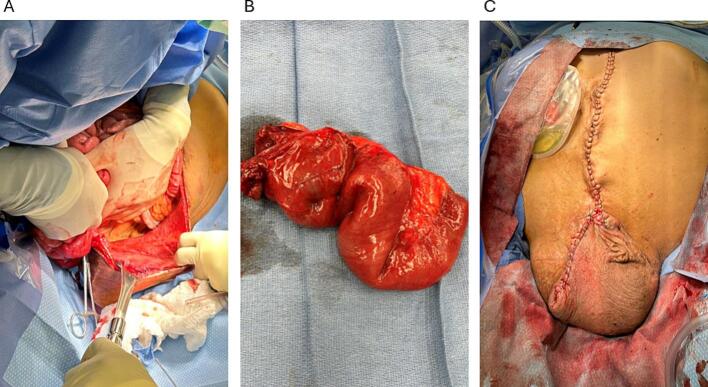
A) Displacement of bowel segments to visualize the scrotal cavity. B) Resected small bowel segment C) Closed incision extending from the midline to the distal scrotum.

The incision was extended toward the scrotum, encompassing the fistula orifice while preserving a skin patch attached to the fistulized bowel (Fig. 4A). The colon was intact, with the ileal conduit originating near the ileocecal junction. The fistulized ileal loop was located mid-small bowel, with 2 m of normal bowel proximal and nearly 1 m distal. The affected bowel segment was resected while preserving more than 3 m of viable small bowel (Fig. 4B).

The resulting enterotomy, anastomosis, and mesenteric defect were closed with interrupted PDS 3/0 sutures. Due to limited abdominal space, the bowel loops were repositioned into the scrotum, as the abdominal and scrotal cavities were no longer separated. Minor adhesiolysis and widening of the anterior aspect of the hernia ring were performed to facilitate safe repositioning, improve bowel mobility, and prevent constriction around the anastomosis. The scrotal cavity was lined by peritoneum from the hernia sac, with the right testicle visible along the scrotal sidewall. Following extensive saline irrigation, the wound was closed in two layers without drain placement (Fig. 4C).

**Fig. 5:**
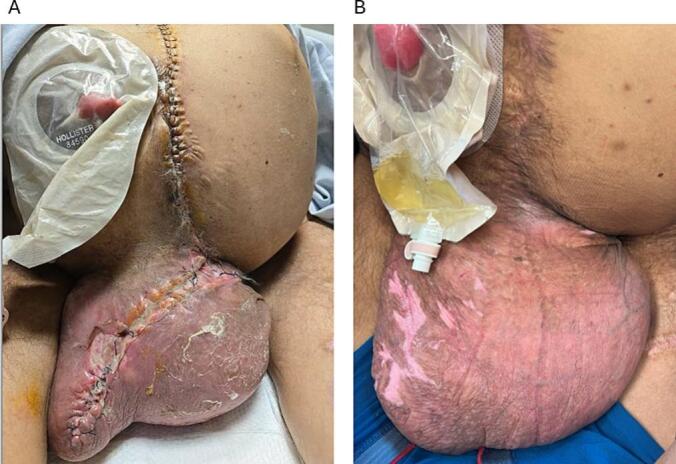
A) Partial wound dehiscence visualized on the scrotal incision on post-operative day 10. B) Healed incision at six-month follow-up.

Postoperatively, scrotal edema developed and raised concern for scrotal dehiscence (Fig. 5A). He was positioned on an air-fluidized bed, received local wound care, and a betadine dressing was applied to the incision daily. Edema gradually subsided, and sutures remained intact. The patient transitioned to an oral diet and demonstrated significant clinical improvement. At six-month follow-up, the wound was closed and the scrotum soft to palpation (Fig. 5B). The patient reported no additional complaints and returned to having regular bowel movements.

## 3 Discussion

GIH is rare, comprising approximately 5 % of inguinal hernia cases, with only 12.5 % involving bilateral GIH [15]. Most reported cases occur in patients with decades-long hernia chronicity and minimal comorbidities [2]. To the best of the authors’ knowledge, this is the first documented case of bilateral GIH in a paraplegic patient with severe lordoscoliosis causing LOD.

LOD is a key concern in the reduction of GIHs and can lead to the development of IAP due to the forced reduction of the herniated viscera into an abdominal cavity that has adapted to being empty [11]. Elevated IAP can then suddenly increase intrathoracic pressure, causing respiratory decompensation with high mortality.

A variety of techniques exist to overcome IAP, but there is no consensus as to which is preferred. Preoperative progressive pneumoperitoneum (PPP) involves the injection of air on a daily schedule to gradually increase the abdominal space and accommodate the respiratory system to increased pressure [11]. Botulinum toxin type A (BTA), a neurotoxin used to induce the flaccid paralysis of the abdominal muscles, can be used alone or synergistically with PPP to increase abdominal volume and improve fascial closure [16]. However, these techniques are typically employed in patients with minimal comorbidities and require a planned, preoperative schedule [17]. Stoppa's technique, a well-established approach to large, bilateral inguinal hernia repair that involves the placement of a large prosthetic mesh in the preperitoneal space, has demonstrated low recurrence rates and minimal postoperative complications [13,18]. More recently, the inversion of the rectus abdominis muscle has been proposed as a novel technique to reduce IAP by rendering the abdominal wall hypotonic, facilitating tension-free closure [19].

In this case, the surgical team encountered a wide, preexisting communication between the abdominal cavity and the scrotum intraoperatively. Favorably, adhesions were minimal, and spontaneous accommodation of bowel within the scrotal sac allowed for tension-free abdominal closure without further reduction. Rather than forcibly reduce the contents and risk an increase in IAP, the scrotal sac was preserved as a functional extension of the abdominal cavity—an approach unique to the patient's anatomical constraints and akin in principle to the “anatomical silo” originally described by Hodgkinson and McIlroth, who created a neoscrotum using resected scrotal skin and a cloverleaf flap to accommodate reduced viscera [1]. A total colectomy and left-sided ileostomy were also considered as a means of decompressing the abdominal cavity, but the patient's primary complaint, in the context of his chronic immobility, was managing both his ileostomy and high ECF output. Creating an additional stoma would have compounded this burden. In the absence of obstruction, a major bowel resection was therefore not pursued.

Scrotal edema is a known complication in laparoscopic inguinal hernia repair, but it is not typically discussed in the setting of GIH [20]. In this patient, scrotal edema resulted in mild incisional separation, warranting daily dressing changes with betadine. Given the patient's paraplegia, offloading the scrotum throughout his hospital stay and positioning the patient during dressing changes was an important consideration to mitigate pressure injury occurrence. Air fluidized beds, which have been utilized for pressure injury prevention in nursing home and post-operative settings, as well as novel techniques for scrotal support, were critical to a complication-free recovery period and may be of benefit to patients with similar anatomical considerations.

## 4 Conclusion

Ultimately, utilizing the scrotum as anatomical neo peritoneum represents a viable, albeit tenuous option for patients with loss of domain in bilateral GIH that requires surgical expertise, multimodal management, and close monitoring postoperatively.

## Author contribution

Hassan, C and Giulianotti, PC were responsible for surgical care of the patient. Ennis, WJ and Knott, JL assisted with post-operative care in a multidisciplinary team with Hassan C, and Giulianotti, PC. Knott, JL drafted the case report, and all other authors offered valuable guidance and feedback throughout the writing and editing process.

## Consent

Written informed consent was obtained from the patient for publication of this case report and accompanying images.

## Guarantor

Hassan, C.

## Sources of funding

This research did not receive any specific grant from funding agencies in the public, commercial, or not-for-profit sectors.

## Declaration of competing interest

William J. Ennis is a consultant for Healogics, LLC. Pier Cristoforo Giulianotti has a consultant agreement with Covidien/Medtronic and an institutional agreement (University of Illinois at Chicago) for training with Intuitive. He is also the Founder and Honorary President of the Clinical Robotic Surgery Association. Chandra Hassan is a proctor for Intuitive and Medtronic. Jack Knott does not declare any interests.
